# Elevated TGF β2 serum levels in Emery-Dreifuss Muscular Dystrophy: Implications for myocyte and tenocyte differentiation and fibrogenic processes

**DOI:** 10.1080/19491034.2018.1467722

**Published:** 2018-05-18

**Authors:** Pia Bernasconi, Nicola Carboni, Giulia Ricci, Gabriele Siciliano, Luisa Politano, Lorenzo Maggi, Tiziana Mongini, Liliana Vercelli, Carmelo Rodolico, Elena Biagini, Giuseppe Boriani, Lucia Ruggiero, Lucio Santoro, Elisa Schena, Sabino Prencipe, Camilla Evangelisti, Elena Pegoraro, Lucia Morandi, Marta Columbaro, Chiara Lanzuolo, Patrizia Sabatelli, Paola Cavalcante, Cristina Cappelletti, Gisèle Bonne, Antoine Muchir, Giovanna Lattanzi

**Affiliations:** a Neurology IV – Neuroimmunology and Neuromuscular Diseases Unit, Foundation IRCCS Neurological Institute “Carlo Besta”, Milan, Italy; b Neurology Department, Hospital San Francesco of Nuoro, Nuoro, Italy; c Department of Clinical and Experimental Medicine, University of Pisa, Pisa, Italy; d Cardiomyology and Medical Genetics, Department of Experimental Medicine, Campania University “Luigi Vanvitelli” (former denomination: Second University of Naples), Italy; e Department of Neurosciences “Rita Levi Montalcini”, University of Turin, Turin, Italy; f Institute of Applied Sciences and Intelligent Systems “ISASI Edoardo Caianello”, National Research Council of Italy, Messina, Italy; g Istituto di Cardiologia, Università di Bologna, Policlinico S.Orsola-Malpighi, Bologna, Italy; h Cardiology Division, Department of Diagnostics, Clinical and Public Health Medicine, University of Modena and Reggio Emilia, Policlinico di Modena, Modena, Italy; i Department of Neurosciences, Odontostomatological and Reproductive Sciences, University of Naples “Federico II”, Naples, Italy; j Institute of Molecular Genetics (IGM)-CNR, Unit of Bologna, Bologna, Italy; k Laboratory of Musculoskeletal Cell Biology, Rizzoli Orthopaedic Institute, Bologna, Italy; l Department of Neurosciences, Neuromuscular Center, University of Padova, Padova, Italy; m Istituto Nazionale di Genetica Molecolare “Romeo and Enrica Invernizzi”, Milan, Italy; n Institute of Cell Biology and Neurobiology, IRCCS Santa Lucia Foundation, Rome, Italy; o Sorbonne Universités, UPMC Univ Paris 06, INSERM UMRS974, CNRS FRE3617, Center for Research in Myology, Institut de Myologie, G.H. Pitié Salpêtrière, Paris Cedex 13, France

**Keywords:** Laminopathies, *LMNA* gene, lamin A/C, Emery-Dreifuss Muscular Dystrophy type 2 (EDMD2), Limb-Girdle muscular Dystrophy 1B (LGMD1B), Dilated Cardiomyopathy (CMD1A), Transforming growth factor beta 2 (TGF β2), tendon fibrosis, muscle fibrosis, muscular differentiation

## Abstract

Among rare diseases caused by mutations in *LMNA* gene, Emery-Dreifuss Muscular Dystrophy type 2 and Limb-Girdle muscular Dystrophy 1B are characterized by muscle weakness and wasting, joint contractures, cardiomyopathy with conduction system disorders. Circulating biomarkers for these pathologies have not been identified. Here, we analyzed the secretome of a cohort of patients affected by these muscular laminopathies in the attempt to identify a common signature. Multiplex cytokine assay showed that transforming growth factor beta 2 (TGF β2) and interleukin 17 serum levels are consistently elevated in the vast majority of examined patients, while interleukin 6 and basic fibroblast growth factor are altered in subgroups of patients. Levels of TGF β2 are also increased in fibroblast and myoblast cultures established from patient biopsies as well as in serum from mice bearing the H222P *Lmna* mutation causing Emery-Dreifuss Muscular Dystrophy in humans. Both patient serum and fibroblast conditioned media activated a TGF β2-dependent fibrogenic program in normal human myoblasts and tenocytes and inhibited myoblast differentiation. Consistent with these results, a TGF β2 neutralizing antibody avoided fibrogenic marker activation and myogenesis impairment. Cell intrinsic TGF β2-dependent mechanisms were also determined in laminopathic cells, where TGF β2 activated AKT/mTOR phosphorylation. These data show that TGF β2 contributes to the pathogenesis of Emery-Dreifuss Muscular Dystrophy type 2 and Limb-Girdle muscular Dystrophy 1B and can be considered a potential biomarker of those diseases. Further, the evidence of TGF β2 pathogenetic effects in tenocytes provides the first mechanistic insight into occurrence of joint contractures in muscular laminopathies.

## Introduction

Emery-Dreifuss Muscular Dystrophy (EDMD) is a rare genetic condition causing elbow and Achilles tendon contractures, rigid spine, muscle weakness and wasting and cardiomyopathy with conduction system disorder [1]. Signs of lipodystrophy are observed in a significant percentage of EDMD patients [2,3]. The disease is linked to mutations in nuclear envelope/lamina genes including *LMNA*, *EMD*, *SYNE 1/2* and can be also caused by *FHL1* mutations or worsened by modifiers such as *SUN1* and *SUN2*, also encoding nuclear envelope proteins [4]. We have previously described pathogenetic mechanisms of EDMD by showing that *LMNA* mutations affect lamin A/C levels and phosphorylation [5] and cause clustering of myonuclei, an effect also involving SUN1 and SUN2 [6]. Although muscle weakness and wasting can be in part explained by those mechanisms as well as by altered expression of myogenic genes downstream of altered lamin-dependent chromatin regulation [4,7], those findings can barely account for some off-target effects such as neck adipose tissue hypertrophy, ankle and elbow contractures or for variable severity of disease among patients [1]. In recent studies [8,9], off-target effects in muscular dystrophy have been ascribed to systemic factors. In Duchenne muscular dystrophy mouse models and human tissues, altered transforming growth factor beta 2 (TGF β2) secretion has been reported and linked to either bone defects or muscle fibrosis or both [8, 9]. Moreover, TGF β2-dependent fibrogenic conversion of muscle cell precursors has been demonstrated in the mouse model of Duchenne muscular distrophy (Mdx mice) [9]. Along this line, inhibition of TGF β release by latent TGF β-binding protein ameliorated the dystrophic phenotype in the same mouse model [10]. Altered TGF β signaling has been also reported in laminopathies. In Mandibuloacral Dysplasia, a mild progeroid laminopathy with skeletal and adipose tissue involvement, increase of TGF β2 secretion in cells and serum has been reported and the effect has been linked to activation of the AKT/mTOR pathway leading to production of pro-osteoclastogenic factors and bone resorption activity [11, 12]. Moreover, enhanced AKT and mTOR phosphorylation was observed in Lmna^H222P/H222P P^ mice at 4 weeks of age and phosphorylation further increased with age [13-16]. This effect was associated with autophagy impairment and was rescued by temsirolimus and selumetinib, drugs that activate the autophagic pathway [14]. Of note, in this mouse model, upregulation of TGFbeta 2 mRNA and activation of downstream signaling has been recently demonstrated [13].

In a comprehensive analysis, we have described and critically reviewed the phenotype of a cohort of Italian patients affected by type 2 EDMD (EDMD2), the muscular dystrophy caused by *LMNA* mutations [1]. In the study here reported, samples from those individuals were collected, other samples from laminopathic patients affected by Limb-Girdle muscular Dystrophy 1B (LGMD 1B) were also included and levels of cytokines, chemokines and growth factors were determined in serum and cell culture medium. This paper reports the results of the analysis and shows that TGF β2 is consistently elevated in the vast majority of EDMD2 patients, irrespective of gender and age. Moreover, TGF β2 levels are higher in EDMD2 than in other muscular dystrophies. At the cellular level, TGF β2 increase impairs myogenic differentiation and elicits expression of pro-fibrotic genes both in myoblasts and tenocytes. Moreover, tenocytes cultured in the presence of EDMD2 patient serum show upregulation of tenomodulin, a TGF β2-induced differentiation marker. Our data suggest a TGF β2 neutralizing antibody as a potential pharmacological tool to be explored in animal models and identify circulating TGF β2 as biomarker for EDMD2 and LGMD 1B.

## Results

### TGF β2 increase in EDMD2 serum

**Figure 1.**
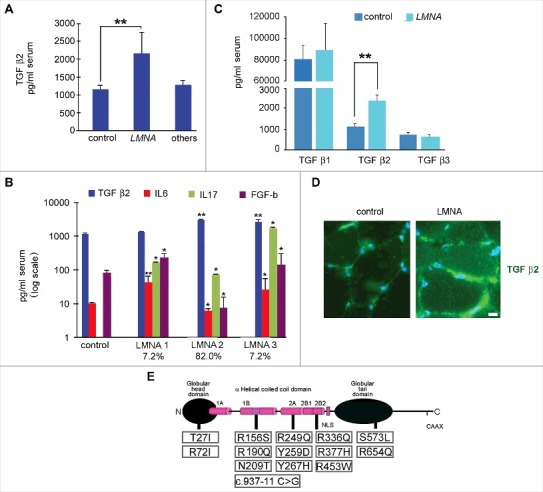
Cytokine level is affected in muscular laminopathies. (A) Levels of TGF β2 in sera of healthy donors (control), patients affected by muscular laminopathies (*LMNA)* and patients affected by other neuromuscular diseases, including Duchenne Muscular dystrophy, Becker muscular dystrophy and Myotonic Dystrophy (others). (B) Levels of TGF β2, IL6, IL17 and FGF-b in sera of controls and *LMNA* symptomatic patients. Based on cytokine expression pattern, sera from *LMNA* symptomatic patients are divided in three subgroups (LMNA1, LMNA2, LMNA3); the percentage of patients in each subgroup out of all examined *LMNA* patients is reported. (C) Levels of TGF β1, TGF β2, TGF β3 in sera of controls and *LMNA* patients. (D) Representative image of immunofluorescence detection of TGF β2 (green) in control and *LMNA* muscle tissue (in this case R190Q/R249Q heterozygous compound mutation in *LMNA* was determined in the EDMD2 patient). Nuclei are stained with DAPI. Bar: 10µm. (E) Scheme of *LMNA* mutations detected in laminopathic patients examined in this study. Means ± standard deviation are shown in graphs. Statistically significant differences are indicated by an asterisk (p<0.05), double asterisk (p<0.01) or triple asterisk (p< 0.001).

The multiple cytokine analysis of EDMD2 and LGMD 1B sera showed that TGF β2 was elevated in the vast majority of affected patients irrespective of gender and age (Fig. 1A). TGF β2 increase was much more significant in EDMD2 than in other neuromuscular diseases including Duchenne Muscular dystrophy (DMD), Becker muscular dystrophy (BMD) and Myotonic Dystrophy (MD) (Fig. 1A). Other cytokines affected in EDMD2 sera were Interleukin (IL) 17 (IL17) that was increased to a variable extent in most patients and basic FGF (FGF-b), although this growth factor was increased in subgroups of patients (LMNA1 and 3, Fig. 1B) and decreased in other patients (LMNA2 group, Fig. 1B). IL6 was increased in two subgroups of patients, the first (LMNA1, Fig. 1B) presenting normal serum levels of TGF β2, the second (LMNA3, Fig. 1B), presenting both TGF β2 and IL6 increase in serum. A third subgroup of patients (LMNA2, Fig. 1B), which represented 82% of all examined cases, showed increased levels of TGF β2 and IL17, while IL6 level was slightly decreased. Moreover, in all EDMD sera, neither TGF β1 nor TGF β3 were significantly affected (Fig. 1C). All patients included in LMNA 1–3 subgroups were symptomatic, presenting muscle weakness and wasting and/or cardiomyopathy with conduction disorders (Table 1). Asymptomatic individuals bearing EDMD2-linked *LMNA* mutations did not show increase of TGF β2 levels and presented normal levels of IL6, while IL17 secretion was moderately increased (Fig. 1S). In muscle biopsies from EDMD2 patients, TGF β2 mean fluorescence intensity was increased in the perimisium (Fig. 1D). Since *LMNA* mutations in the examined patients had been determined all along the protein sequence (Fig. 1E), ruling out the possibility of site-specific effects, these data suggested that either altered cytokine secretion was triggered by the ongoing degenerative process affecting skeletal or cardiac muscle or it was part of the cellular response to mutated *LMNA* expression. The following paragraphs report experiments aimed at evaluating both hypotheses for TGF β2.

**Table 1.** List of patients involved in the study.

Pathology	*LMNA* mutation	Gender	Age	Symptoms	Heart surgery
EDMD 2	c.1007G>A	M	58	Dilated cardiomyopathy, conduction defects, atrial fibrillation	ICD
EDMD 2	c.1007G>A	M	27	Dilated cardiomyopathy, conduction defects, atrial fibrillation	ICD
EDMD 2	c.1007G>A	M	43	Dilated cardiomyopathy, conduction defects, atrial fibrillation	ICD
EDMD 2	c.471C>A	M	35	Dilated cardiomyopathy, conduction defects, atrial fibrillation	ICD
EDMD 2	c.625delA	M	30	Dilated cardiomyopathy, conduction defects, atrial fibrillation	
EDMD 2	c.937-11 c>g (IVS5-11C>G)	F	49	Dilated cardiomyopathy, conduction defects, atrial fibrillation	ICD
EDMD 2	c.937-11 c>g (IVS5-11C>G)	F	20	Cardiomyopathy and conduction defects, atrial fibrillation	
EDMD 2	c.625delA	M	45	Cardiomyopathy and conduction defects	
EDMD 2	c.812T>C	M	30	Cardiomyopathy and conduction defects, atrial fibrillation	
EDMD 2	c.799T>C	F	22	Rigid spine, muscular dystrophy, early signs of cardiac compromise, cardiomyopathy	
EDMD 2	c.799T>C	M	20	Atrial fibrillation, cardiac conduction disease, biatral enlargement	
EDMD2	c.854T>A	M	38	Diffuse muscle weakness, early contractures, atrial fibrillation	
CMD1A	c.1608+1G>T	M	38	Ventricular extrasystoles, dilated cardiomyopathy (slight left ventricular dilation)	
LGMD1B	c.1608+1G>T	M	50	Mild axial and pelvic girdle muscle weakness, bundle branch block, atrial fibrillation, mild left ventricular dilatation	ICD
CMD1A	c.1102_1130dup	M	34	Ventricular tachyarrhythmia, slight left ventricular dilation with preserved systolic function	ICD
LGMD1B	c.1102_1130dup	F	63	Muscle weakness and wasting, contractures, cardiomyopathy	heart transplantation
CMD1A	c.1608+1G>T	F	40	Ventricular extrasystoles, tachyarrhythmias	ICD
LGMD1B	c.IVS5-11C>G (c.937-11C>G)	F	37	Cardiomyopathy and conduction defects, atrial fibrillation	
LGMD1B	c.937-11 c>g (IVS5-11C>G)	M	45	Cardiomyopathy and conduction defects, atrial fibrillation	
LGMD1B	c.937-11 c>g (IVS5-11C>G)	F	41	Cardiomyopathy and conduction defects, atrial fibrillation	
LGMD1B	c.937-11 c>g (IVS5-11C>G)	F	77	Cardiomyopathy and conduction defects, atrial fibrillation	
EDMD 2	c.799T>C	F	45	Cardiomyopathy and conduction defects	
EDMD 2	c.812T>C	M	45	Cardiomyopathy and conduction defects	
EDMD2	UN	M	34	Muscle weakness and wasting	
EDMD2	UN	M	21	Muscle weakness and wasting	
EDMD2	UN	M	23	Muscle weakness and wasting	
EDMD2	UN	M	25	Asymptomatic	
EDMD2	c.1357C>T	F	54	Muscle weakness and contractures, dilated cardiomyopathy, conduction disease	
EDMD2	c.1357C>T	F	21	Muscle weakness and contractures, dilated cardiomyopathy, conduction disease	
LGMD1B	c.1718C>T	F	67	Muscle weakness and wasting	
EDMD2	c.1960C>T	F	43	Axonal Neuropathy	
EDMD2	c.214C>T	F	23	Muscle weakness and wasting	
EDMD2	c.1718C>T	M	64	Iper-CK, myalgia	
EDMD2	c.1718C>T	F	54	Muscle weakness and wasting	
EDMD2	c.1718C>T	M	45	Muscle weakness and wasting	
LGMD1B	c. 1146C>T+c.1698C>T	F	34	Muscle weakness and wasting	
LGMD1B	c.1130G>A	M	35	Muscle weakness and wasting	
EDMD2	c.1130G>A	F	81	Asymptomatic	
LGMD1B	c.1130G>A	M	30	Muscle weakness and wasting	
EDMD2	UN	M	39	Muscle weakness, cardiomyopathy, conduction disease	
EDMD2	UN	M	39	Muscle weakness, cardiomyopathy, conduction disease	
CMD1A	c 357-IG>A (IVSI-IG>A)	M	24	Muscle weakness, dilated cardiomyopathy	
CMD1A	c 357-IG>A (IVSI-IG>A)	F	51	Muscle weakness, dilated cardiomyopathy	
EDMD2	c.775T>G	M	27	Muscle weakness and wasting, contractures, cardiomyopathy	ICD
LGMD1B	c.80C>T	M	59	Muscle weakness and wasting, cardiomyopathy	ICD
LGMD1B	c.80C>T	M	33	Muscle weakness, dilated cardiomyopathy	ICD
LGMD1B	c.80C>T	F	52	Muscle weakness and wasting, cardiomyopathy	ICD
EDMD2	c.80C>T	M	26	Asymptomatic	
LGMD1B	c.80C>T	F	57	Muscle weakness and wasting, cardiomyopathy,	
EDMD2	c.80C>T	M	28	Asymptomatic	

### TGF β2 levels are increased in *Lmna^H222P/H222P^*mice

**Figure 2.**
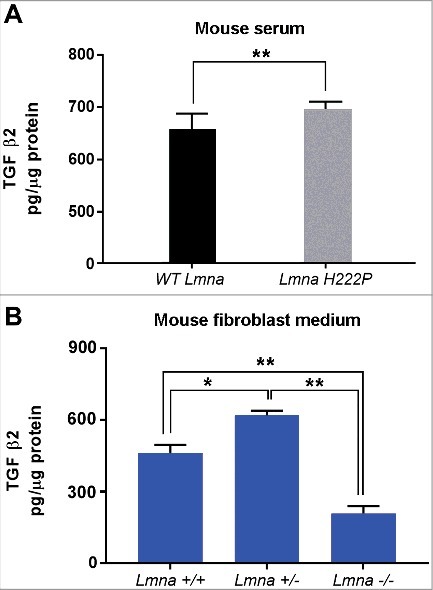
TGF β2 level is increased in laminopathic mice. (a) Levels of TGF β2 in sera of WT mice (n = 6) and *Lmna^H222P/H222P^* mice (*Lmna^H222P^*) (n  = 5). (b) Levels of TGF β2 secreted by fibroblasts isolated from WT mice (*Lmna* ^+/+^), mice bearing heterozigous (*Lmna* ^+/−^) or homozygous (*Lmna* ^−/−^) null mutation in *Lmna* gene and maintained in culture for 5 days. Means ± standard deviation are shown in graphs. Statistically significant differences are indicated by an asterisk (p<0.05) or double asterisk (p< 0.01).

Elevated TGF β2 secretion has been linked to activation of AKT/mTOR signaling and neutralization of TGF β2 has been shown to restore altered AKT/mTOR signaling in cells bearing *LMNA* mutations [11]. Activation of the same signaling pathway has been also reported in *Lmna^H222P/H222P^*mice that also showed transcriptional activation of TGF β2 [13, 14]. Here, we evaluated serum levels of TGF β2 in *Lmna^H222P/H222P^*mice^13^ and *Lmna* null mouse cells [17] (Fig. 2A). TGF β2 was elevated in serum from *Lmna^H222P/H222P^* mice (Fig. 2A), consistent with the reported mRNA increase previously shown in this mouse model [13]. Using *Lmna* null mouse cultured fibroblasts we obtained an unexpected result. In fact, compared to *Lmna* ^+/+^ cell culture medium, TGF β2 levels were significantly increased in *Lmna* ^+/-^ and decreased in *Lmna* ^−/−^ cell culture medium (Fig. 2B). These results could indicate that the presence of non-functional lamin A/C is necessary to trigger TGF β2 secretion, as previously reported in other laminopathic models [11].

### TGF β2 induces a fibrogenic process in myoblasts

**Figure 3.**
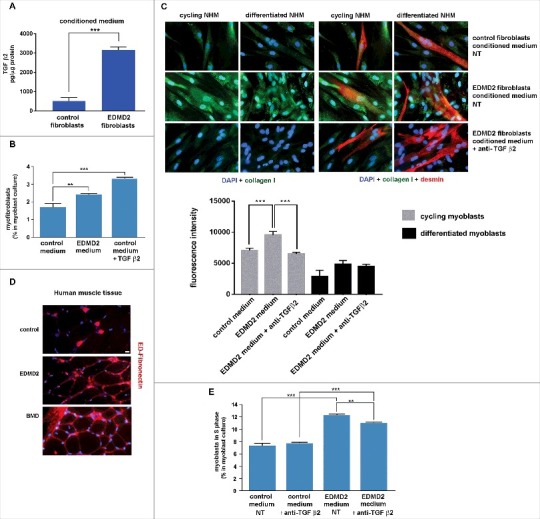
EDMD2 fibroblast medium induces TGF β2-dependent fibrogenic conversion in NHM cultures. (A) TGF β2 secretion in control and EDMD2 fibroblast culture medium. (B) Quantification of myofibroblasts in cultures of control human myoblasts maintained in medium conditioned by control (control) or EDMD2 (EDMD2) fibroblasts or treated with TGF β2 (control + TGF β2). The number of myofibroblasts was determined by counting alpha-SMA positive mononucleated cells (200 cells/sample were counted in three independent experiments). (C) Immunofluorescence staining of collagen I (green) and desmin (red) in cycling and differentiated NHM cultured in presence of medium conditioned by control or EDMD2 fibroblasts, treated (anti-TGFβ2) or not (NT) with anti-TGF β2 neutralizing antibodies. Nuclei were stained with DAPI. Bar: 10µm. (D) Immunofluorescence staining of ED-fibronectin (red) in cryosections of muscle tissue isolated from healthy donors (control), EDMD2 patients (EDMD2) or Becker muscular dystrophy patients (BMD). Nuclei were stained with DAPI. BMD tissue was used as positive control. In control muscle, ED-fibronectin is restricted to the area surrounding blood vessels. Bar: 10µm. (E) Quantitative analysis of proliferating cells in NHM cultures maintained in medium conditioned by control or EDMD2 fibroblasts, treated (anti-TGF β2) or not (NT) with anti-TGF β2 neutralizing antibodies. The number of proliferating cells was determined by flow cytometry. Means ± standard deviation are shown in graphs. Statistically significant differences are indicated by double asterisk (p<0.01) or triple asterisk (p< 0.001).

Elevated TGF β2 levels were not only detected in EDMD2 serum, but also in EDMD2 fibroblast culture media (Fig. 3A). Thus, we hypothesised a double effect of lamin A/C mutations on muscle functionality, caused by local and systemic TGF β2 increase. To confirm the parabiotic effect of TGF β2 on human muscle cells, we co-cultured normal human myoblasts (NHM) with EDMD2 fibroblasts. Cell culture conditioning increased the number of myofibroblasts, as determined by counting alpha-smooth muscle actin (alpha-SMA) and ED-fibronectin [18] positive (double-labelled) cells (Fig. 3B). These results indicated activation of a fibrogenic process. Moreover, intracellular collagen I was increased in conditioned myoblasts [9] (Fig. 3C), demonstrating that a fibrogenic process could be stimulated by EDMD2 fibroblasts in NHM. Differentiating myoblasts conditioned by control cells did not show collagen I staining [9] (Fig. 3C). Conversely, EDMD2 medium induced upregulation of collagen I in cycling myoblasts and even in myogenin-positive committed myoblasts, but not in myotubes (Fig. 3C). To establish the involvement of TGF β2 in that mechanism, we inhibited growth factor activity by a specific neutralizing antibody [19]. Neutralization of TGF β2 reduced collagen I production to levels comparable to controls (Fig. 3C) indicating that the fibrogenic process was mostly driven by TGF β2 signaling. In agreement with those findings, we observed that the fibrogenic marker ED-fibronectin (Fig. 3D) was increased in muscle fibers from EDMD2 patients, though did not reach the high levels observed in BMD muscle (Fig. 3E). These results showed that collagen I-positive myogenic cells were formed in the presence of TGF β2 and contributed to dysregulation of the extracellular matrix in muscle tissue. To test the effect of TGF β2 on cellular proliferation, we analyzed the cell cycle in the presence or not of conditioning media. Myoblast proliferation was stimulated by EDMD2 medium, while neutralization of TGF β2 slightly reduced the proliferation rate (Fig. 3E). This result suggested that the EDMD2 secretome stimulated myoblast proliferation at in least in part through TGF β2.

### TGF β2 impairs myoblast differentiation

**Figure 4.**
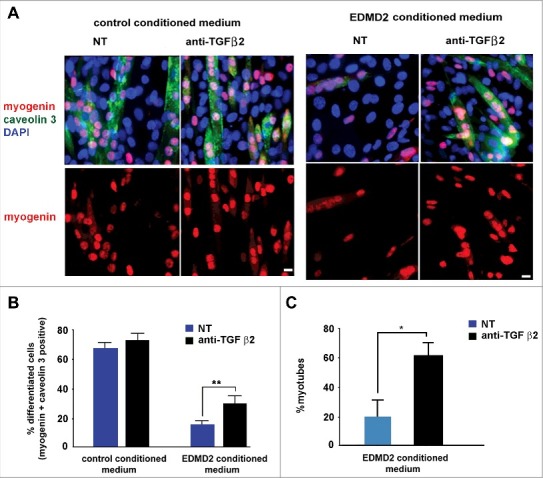
EDMD2 fibroblast medium inhibits differentiation of NHM through TGF β2. (A) Immunofluorescence staining of myogenin (red) and caveolin 3 (green) in C2C12 myoblasts cultured in presence of control or EDMD2 fibroblast medium. Data from samples left untreated (NT) or treated with anti-TGF β2 antibody (anti-TGF β2) are reported. Nuclei are counterstained with DAPI. Bar: 10µm. (B) Percentage of differentiated cells in C2C12 myoblast cultures conditioned by control or EDMD2 medium. Data from samples left untreated (NT) or treated with anti-TGF β2 antibody (anti-TGF β2) are reported. (C) Percentage of differentiated NHM conditioned by EDMD2 medium. Data from samples left untreated (NT) or treated with anti-TGF β2 antibody (anti-TGF β2) are reported. Means ± standard deviation are shown in graphs. Statistically significant differences are indicated by an asterisk (p<0.05) or double asterisk (p< 0.01).

Increased proliferation of muscle precursors could affect myogenic differentiation rate, contributing to muscular dystrophy [20]. To test the hypothesis that TGF β2 could contribute to altered myoblast differentiation in EDMD2, we explored the effect of the EDMD2 secretome on myotube formation. Mouse C2C12 myoblast differentiation was impaired to a significant extent by EDMD2 fibroblast culture medium and could be rescued by neutralization of TGF β2 activity as demonstrated by counting cells positive for myogenic markers [6] including myogenin and caveolin 3 (Fig. 4A, B). Also in NHM treated with EDMD2 medium, differentiation rate was significantly reduced in a TGF β2-dependent way (Fig. 4C). These data demonstrated that TGF β2 levels affect the turnover of muscle cells and the myogenic process can be re-activated by its blockade.

### TGF β2 affects tenocyte differentiation favoring fibrosis

**Figure 5.**
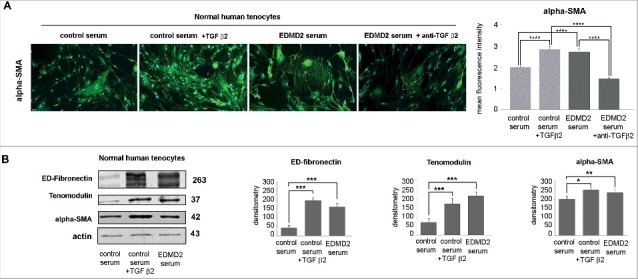
TGF β2 from EDMD2 serum induces fibrosis markers in normal human tenocytes. (A) Immunofluorescence staining of alpha-SMA in normal human tenocytes cultured in the presence of control serum, control serum + TGF β2, EDMD2 serum or EDMD2 serum + anti-TGF β2. Nuclei were counterstained with DAPI. Bar, 20 μm. Quantitative analysis of mean fluorescence intensity of alpha-SMA is reported in the graph. (B) Western blot analysis of ED-fibronectin, tenomodulin and alpha-SMA in control tenocytes exposed to control serum, control serum + TGF β2 or EDMD2 serum. Densitometric analysis of immunoblotted bands is reported in the graphs. Means ± standard deviation are shown in graphs. Statistically significant differences are indicated by an asterisk (p<0.05), double asterisk (p< 0.01) or triple asterisk (p< 0.001).

Tenocytes represent possible effectors of EDMD2 pathogenesis, since contractures of the Achilles tendons and elbows are the first sign of disease in most affected individuals [1]. Here, we tested the hypothesis that elevated TGF β2 levels might interfere with tenocyte differentiation and induce a fibrotic phenotype. TGF β2 is an inducer of collagen I in tenocytes and the process contributes to differentiation [21,22]. However, an amount of TGF β2 comparable to that observed in patient serum caused a striking increase of alpha-SMA, a marker of myofibroblasts, in control tenocytes (Fig. 5A). Moreover, in control tenocytes cultured in the presence of EDMD2 serum, conversion to myofibroblasts, labeled by alpha-SMA was observed and alpha-SMA overall fluorescence intensity was significantly increased (Fig. 5A). Importantly, neutralization of TGF β2 reduced the rate of fibrogenic conversion, as shown by reduced alpha-SMA positivity (Fig. 5A).

As expected [22], TGF β2-treated tenocytes expressed high tenomodulin levels (Fig. 5B). The same effect was elicited by culturing tenocytes in EDMD2 serum (Fig. 5B). Thus, EDMD2 serum triggered the expression of a late tenocyte differentiation marker by mimicking the effect of TGF β2.

The whole evaluation of the above reported data led us to the conclusion that excess levels of TGF β2 drive fibrotic processes in EDMD2 tendons and muscle cells, while also enhancing tenocyte differentiation.

### TGF β2 signaling in EDMD2

**Figure 6.**
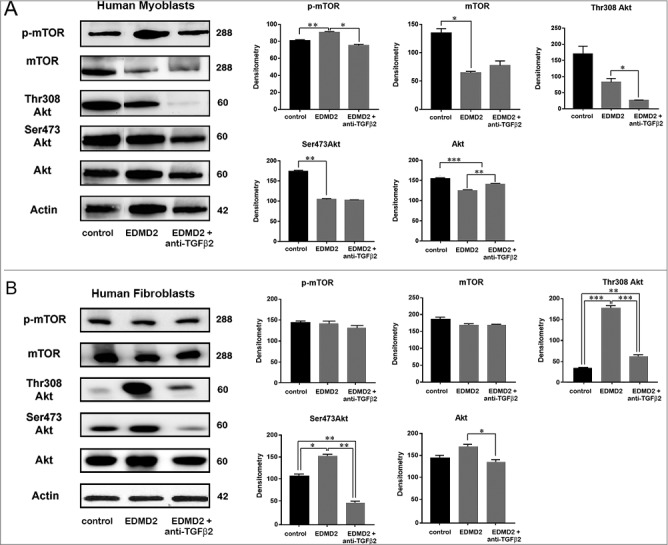
TGF β2 affects the AKT/mTOR pathway in EDMD2. Western blot analysis of p-mTOR, mTOR, Thr308 Akt, Ser473 Akt, Akt and actin performed in (A) control myoblasts (control), EDMD2 myoblasts (EDMD2) and EDMD2 myoblasts treated with anti-TGF β2 neutralizing antibody (EDMD2 + anti-TGF β2) or (B) control fibroblasts (control), EDMD2 fibroblasts (EDMD2) and EDMD2 fibroblasts treated with anti-TGF β2 neutralizing antibody (EDMD2 + anti-TGF β2). Actin bands are shown as protein loading control. Molecular weight markers are reported in kDa. Densitometric analysis of immunoblotted bands normalized on actin is reported in the graphs. Means ± standard deviation are shown in graphs. Statistically significant differences are indicated by an asterisk (p<0.05), double asterisk (p< 0.01) or triple asterisk (p< 0.001).

To test the signalling pathway downstream of TGF β2, we examined control and EDMD2 myoblast lysates by western blot analysis. Statistically significant increase of mTOR phosphorylation was determined in EDMD2 myoblasts (Fig. 6A). However, in fibroblasts from EDMD2 subjects, mTOR phosphorylation was not affected, whereas Akt was activated, as demonstrated by increase of serine 308 and serine 473 phosphorylation (Fig. 6B). Importantly, neutralization of TGF β2, obtained by adding the specific antibody in cell cultures, reduced mTOR phosphorylation in myoblasts and AKT phosphorylation in fibroblasts (Fig. 6). These results showed that increased TGF β2 secretion elicits AKT/mTOR activation in EDMD2 cells through diverse branches of the signalling pathway in diverse cell types.

## Discussion

The reported study provides several advances in the understanding of EDMD2 pathogenesis. 1) Increase of TGFbeta 2 secretion is identified as a hallmark of disease both in patient serum and in animal models as well as in cell cultures derived from patient muscle biopsies. 2) Activation of AKT/mTOR pathway is demonstrated as an effect of increased TGFbeta 2 signaling in EDMD2 myoblasts. 3) Fibrogenic response to TGF beta 2 secretion is determined both in human myoblasts and tenocytes. 4) Tenocytes are recognised as a relevant target of disease in EDMD2, based on their response to EDMD2 secretome stimulus. 5) Neutralizing TGF beta 2 antibodies are indicated as a potential therapeutic tool to be further explored in preclinical models.

The consistent increase in TGFbeta 2 secretion determined in EDMD2 patients, irrespective of mutated *LMNA* sequence, gender and age of patients, allows us to propose TGFbeta 2 as the first circulating biomarker of this disease. Of note, TGFbeta 2 levels in EDMD2 were compared to those measured in DMD serum [8] and found to be significantly higher. This finding hints to a specific effect of *LMNA* mutations on TGFbeta 2 regulation that is not fully elucidated. Our previous data obtained in primary cells and experimental models of progeroid laminopathies, show that mutated lamin A/C fails to downregulate this cytokine, while wild-type lamin A does repress TGF beta 2 expression [11]. We further showed that secreted TGFbeta 2 is able to activate Akt phosphorylation in osteoblast-like cells and fibroblasts [11] leading to degradation of lamin A itself [11,23–25]. Thus, we suggest that lamin A-TGFbeta 2 interplay could contribute to reduce *LMNA* expression in the presence of mutated lamin A. This hypothesis and the possibility that lamin A might in turn influence TGF beta 2 expression at the transcriptional level warrant further investigation.

In this study, we show that the mTOR pathway is activated in EDMD2 myoblasts and the mechanism is dependent on TGFbeta 2, as demonstrated by the inhibitory effect exerted by neutralizing TGFbeta 2 antibodies. Of note, we found a different response to TGF beta 2 secretion in fibroblasts versus myoblasts even from the same affected individual. Akt serine 473 and 308 phosphorylation was in fact increased in fibroblasts in a TGF beta -2 dependent fashion, but not in myoblasts, while mTOR phosphorylation was selectively increased in myoblasts. This results could suggest that mTOR activation in myoblasts triggers mTORC1-dependent Rictor phosphorylation that inhibits mTORC2-mediated Akt phosphorylation [26]. Conversely, direct activation of Akt, possibly dependent on Erk 1/2 phosphorylation [13], likely occurs in EDMD2 fibrobalsts, as determined in other laminopathic fibroblasts [11] and in mouse models [13].

A plethora of effects may potentially derive from Akt/mTOR activation. Increased mTOR phosphorylation, here determined in EDMD2 myoblasts, is expected to impair the autophagic process, as previously reported in animal models of EDMD2 by Ramos et al [15]. and Choi et al [14]. Here, we did not investigate changes in autophagic processes, an issue that will be addressed in further studies. Rather, we focused on the effect elicited by TGF beta 2 on the fibrogenic process. Upstream of fibrogenic mechanisms, both mTOR activation and mTOR-independent Akt phosphorylation may occur [13,27]. In fact, simultaneous activation of the mTOR subunits mTORC1 and mTORC2 has been shown to mediate TGF beta 2-dependent upregulation of α-SMA and myofibroblast formation [27]. On the other hand, collagen I and connective tissue growth factor (CTGF) upregulation related to Akt hyperactivation has been demonstrated in laminopathic *Lmna*^H222P/H222P^ mice [13,28]. Thus, it is tempting to speculate that different branches of TGFbeta 2 signaling pathway contribute in a cell/tissue-specific way to the onset of fibrosis in EDMD2. A demonstration of engagement of mTOR in a tissue-specific way has been reported in response to insulin stimulus [29] and different levels of proteins of the Akt/mTOR pathway have been found in human tissues [30].

Fibrosis was previously determined in mouse models of EDMD2, especially in myocardium [13]. Moreover, several reports documented fibrotic areas in muscle and heart of EDMD2 patients [31-33]. Here, we provide a mechanistic explanation of this phenomenon by showing involvement of TGFbeta 2. Moreover, we identify for the first time a major role of TGFbeta 2 in the activation of fibrotic process in tenocytes. Although tenocytes from EDMD2 individuals were not available, our data show that EDMD2 cell culture medium or serum from laminopathic patients strongly increase both levels of profibrotic factors alpha-SMA and collagen I and expression of the late tenocyte marker tenomodulin [22]. The major role of TGFbeta 2 on tendon progenitor cell recruitment, collagen I and tenomodulin upregulation and tenocyte differentiation is well documented [22,34]. The condition we envisage in EDMD2 may affect tendon functionality by inducing both dysregulated differentiation and formation of myofibroblasts, typical of fibrotic tissue. An interesting clinical observation in EDMD2 is that contractures are in most cases a first symptom of disease, often preceding muscle weakness and wasting [1]. Our finding that TGFbeta 2 increase is a common trait of symptomatic patients affected by muscular laminopathies, even at early stages of disease progression, may in part explain the early occurrence of tendon contractures. In this respect, it is worth mentioning data obtained in a mouse model that demonstrate different response of tenocytes to TGFbeta 2 stimulus, depending on development stage and/or mechanical loading [22]. Thus, it is possible that elevated TGFbeta 2 secretion elicits tenocyte defects and contributes to contractures in a defined period of tendon development. This is consistent with the observation that many EDMD2 patients undergo Achilles tendon lengthening to resolve contractures and toe walking at a very young age and do not experience any further problems in the following years. This issue warrants investigation in view of potential pharmacological treatments.

Both in muscle cells and tenocytes, we show that a neutralizing TGFbeta 2 antibody may reduce fibrogenic markers. To which extent this antibody could be exploited to counteract in vivo fibrosis and altered cellular differentiation must be assessed in preclinical studies. So far, we cannot rule out the possibility that high TGFbeta 2 levels in laminopathic patients might be protective through the mTOR-dependent pathway leading to degradation of mutated lamin A^11^.

In support of this possibility, we recently observed that patients affected by the most severe form of muscular laminopathy, the *LMNA*-related congenital muscular dystrophy (L-CMD), show an overall altered cytokine profile, but normal levels of TGFbeta 2.

Overall, in the study here reported, TGFbeta 2 emerges as a biomarker of disease for EDMD2 and LGMD1B to be further explored as therapeutic target.

## Materials and methods

### Patients

Fifty EDMD2 and LGMD1B patients were involved in this study. Information on *LMNA* mutation, phenotype, sex and age of patients at collection of biological samples is reported in Table 1. All patients gave their written informed consent and the study was approved by the ethical Committee of the Fondazione Neurologica Carlo Besta in Milan that coordinated secretome analysis.

### Serum samples

Serum samples were collected from previously characterized EDMD2 and LGMD1B patients (Table 1). Serum from 22 healthy donors (3 males and 19 females, age range: from 23 to 50 years) was also tested. Frozen serum samples were gathered and subjected to multiple cytokine screening at the Fondazione Neurologica Carlo Besta Institute.

### Animal samples

Serum samples were collected from *Lmna*^H222P/H222P^ mice fed chow and housed in a disease-free barrier facility with 12 h/12 h light/dark cycles. Blood collection was performed according to Ministère de l′Éducation Nationale de l'Enseignement Supérieur et de la Recherche France at Center for Research in Myology Paris.

### Cell culture and treatments

Human myoblast and fibroblast cultures were obtained from healthy donors, LGMD1B patients carrying the Y259D *LMNA* mutation [6] or EDMD2 patients bearing the H506P *LMNA* mutation [35]. Tenocytes were established from biopsies of healthy donors undergoing orthopaedic surgery. Muscular tissue was from biopsies of healthy donors, patients affected by EDMD2 (*LMNA* H506P mutation) or Becker muscular dystrophy (BMD). These samples belong to the BioLaM biobank hosted at Rizzoli Orthopaedic Institute-CNR Institute of Molecular Genetics in Bologna. All samples were obtained following written consent and according to Italian and EU rules. Fibroblast cultures from adult *Lmna^+/+^*, Lmna^+/−^ and Lmna^−/−^ mice were established and characterized at the CNR Institute of Cellular Biology and Neurobiology, according to approved experimental protocols. Mouse C2C12 cells from the European Cell Culture Collection were purchased from Sigma.

Human myoblasts and fibroblasts were grown in Dulbecco's modified Eagle's medium – High Glucose (DMEM-HG), supplemented with 20% fetal bovine serum (FBS) and antibiotics mix, at 37°C, 5% CO2. C2C12 myoblasts were grown in DMEM supplemented with 10% FBS, at 37°C and 5% CO2. Human control myoblasts plated in 12-well tissue culture plates were cultured in the presence of conditioned medium from healthy donor (control) or EDMD2 fibroblasts.

In order to neutralize TGF β2, human myoblast and C2C12 cultures were treated for 72 hours with human TGF β2 neutralizing antibody (ab66045, Abcam, Cambridge, UK) at the concentration of 0,03 μg/ml or mouse TGF β2 neutralizing antibody at the concentration of 0,01 μg/ml (MAB7346, R&D System, Abington, UK), respectively.

Control tenocytes were cultured in the presence of serum from healthy donors (control) or EDMD2 patients. Moreover, TGF β2 was added to some tenocyte cultures at the concentration of 1 ng/mL for 72 hours [34].

### Western blot analysis

Whole cell lysates were prepared in RIPA buffer (20 mM Tris-HCl, pH 7.0, 1% Nonidet P-40, 150 mM NaCl, 10% glycerol, 10 mM EDTA, 20 mM sodium fluoride, 5 mM sodium pyrophosphate, 1 mM Na3VO4, 1 mM PMSF, 10 μg/ml leupeptin and 10 μg/ml pepstatin) by incubation at 4°C for 30 minutes. Protein concentration of samples was determined using the Bradford protein assay (Bio-Rad Laboratories, Mitry-mory, France). 50 µg of protein lysate were diluted in sample buffer, subjected to SDS-PAGE and transferred to nitrocellulose membrane (BioRad). Membranes were saturated with 4% BSA and incubated with primary antibodies overnight at 4°C. Secondary antibodies were incubated for 1 hour at room temperature. Immunoblotted bands were revealed by enhanced chemiluminescence (Amersham ECL detection system, GE Healthcare). Intensity measurement was performed using a BioRad densitometer (GS 800) equipped with Quantity One Software. Actin was used as a protein loading control for quantitative analysis.

### Immunofluorescence

Myoblasts grown on coverslips were fixed with 4% parafolmaldehyde at 4°C and permeabilized with 0.15% TRITON X-100 at room temperature. Non-specific binding was avoided by saturating samples with 4% BSA in PBS. Coverslips were then incubated overnight at 4°C with primary antibody and 1hour at room temperature with secondary antibody and counterstained with DAPI. Samples were analyzed with a Nikon Eclipse Ni fluorescence microscope using the NIS AR software. All images were taken at similar exposures within an experiment for each antibody. Images were processed using Adobe Photoshop 7 (Adobe Systems).

### Antibodies

Primary antibodies for immunoblotting and/or immunofluorescence were goat polyclonal alpha-SMA (ab5694, Abcam, Cambridge, UK), mouse monoclonal anti-fibronectin (ab6328, Abcam, Cambridge,UK), rabbit polyclonal anti- myogenin (sc576, Santa Cruz Biotechnology, Heidelberg, Germany), rat monoclonal anti-caveolin 3 (BD 610421, BD Transduction Laboratories, USA), rabbit polyclonal anti-TGF β2 (sc90, Santa Cruz Biotechnology, Heidelberg, Germany), rabbit polyclonal anti-collagen I antibody (ab34710, Abcam, Cambridge, UK), anti-desmin (MA1-22150, Sigma), anti-tenomodulin (ab81328, Abcam, Cambridge, UK), anti-actin (Santa Cruz).

### Cytometry

The percentage of cells in the G0/G1, S and G2/M phase of the cell cycle was determined by the Muse Cell Analyzer (Merck Millipore, France) using Muse Cell Cycle Kit following manufacturer's instructions.

### Quantification of cytokine levels in sera and culture media

Sera derived from EDMD2 patients and from healthy donors were analyzed using Bio-Plex Pro Human 27-plex Assay kit (Bio-Rad Laboratories) and TGF β 3-plex kit (Bio-Rad Laboratories, Mitry-mory, France) following manufacturer's instructions. TGF β2 secretion in culture media was measured by ELISA according to the protocol supplied by the manufacturer (Bio-Rad Laboratories, Mitry-mory, France).

### Statistical analysis

Statistical analysis was performed using Graphpad software (Prism, California, USA). Student's t test was used for comparisons between two groups and one-way ANOVA with Tukey's post hoc test was used for comparisons between multiple groups. Significance was accepted at *p* < 0.05 and data are presented as mean ± standard deviation of the mean.

## Supplementary Material

Suppl_Material.zip
